# K-mer-Based Motif Analysis in Insect Species across *Anopheles*, *Drosophila*, and *Glossina* Genera and Its Application to Species Classification

**DOI:** 10.1155/2019/4259479

**Published:** 2019-11-15

**Authors:** Matyas Cserhati, Peng Xiao, Chittibabu Guda

**Affiliations:** Department of Genetics, Cell Biology & Anatomy, University of Nebraska Medical Center, Omaha, NE 68198, USA

## Abstract

Short k-mer sequences from DNA are both conserved and diverged across species owing to their functional significance in speciation, which enables their use in many species classification algorithms. In the present study, we developed a methodology to analyze the DNA k-mers of whole genome, 5′ UTR, intron, and 3′ UTR regions from 58 insect species belonging to three genera of *Diptera* that include *Anopheles*, *Drosophila*, and *Glossina*. We developed an improved algorithm to predict and score k-mers based on a scheme that normalizes k-mer scores in different genomic subregions. This algorithm takes advantage of the information content of the whole genome as opposed to other algorithms or studies that analyze only a small group of genes. Our algorithm uses k-mers of lengths 7–9 bp for the whole genome, 5′ and 3′ UTR regions as well as the intronic regions. Taxonomical relationships based on the whole-genome k-mer signatures showed that species of the three genera clustered together quite visibly. We also improved the scoring and filtering of these k-mers for accurate species identification. The whole-genome k-mer content correlation algorithm showed that species within a single genus correlated tightly with each other as compared to other genera. The genomes of two *Aedes* and one *Culex* species were also analyzed to demonstrate how newly sequenced species can be classified using the algorithm. Furthermore, working with several dozen species has enabled us to assign a whole-genome k-mer signature for each of the 58 Dipteran species by making all-to-all pairwise comparison of the k-mer content. These signatures were used to compare the similarity between species and to identify clusters of species displaying similar signatures.

## 1. Introduction

DNA k-mers are short recurring elements in the genomes of all living species. These elements are both conserved and diverged across species owing to their functional significance, which enables these k-mer signatures ideal for species identification. Several recent studies have described the distribution of statistically significant k-mers in the genomes and several regulatory subregions (core, proximal, distal promoters, and 3′ and 5′ UTRs) in a small number of plant species as well as modern and archaic humans [1–3]. A k-mer is a type of short oligonucleotide of length k. K-mers can be part of core segments of transcription factor binding sites or regulatory elements that take part in protein binding and gene regulation in different subregions of the genome.

The present version of the algorithm is an alignment-free k-mer sequence comparison method. Such methods involve statistical analysis and comparison of k-mers between the genomes of two species. These methods vary in the statistical measures applied, such as the comparison of word frequency, incorporation of information theory, universal sequence maps, and the measurement of complexity [4]. The advantages of k-mer-based alignment-free methods over alignment-based phylogenetic algorithms are that they can process the data much faster and eliminate biases that could be induced by using *a priori*-defined guide trees when performing the alignment, and subjective selection of alignment scoring parameters, such as gap opening and extension [5, 6].

Related methods include metagenomic algorithms that are capable of identifying bacterial taxonomic groups based on metagenomic sequence data. Such methods focus on taxon identification but not taxon comparison. Such an algorithm is PhyloPythia, which applies a multiclass support vector machine (SVM) using relative frequency profiles of short oligonucleotides to classify genome fragments as short as 1 Kbp into taxonomic ranks between genus and phylum with high specificity [7]. Another method, TACOA, uses abundance profiles to represent whole-genome sequences and uses a k-nearest-neighbor-classification-based method [8]. It correctly classified fragments larger than 800 bp between 39% and 76% at the genus or superkingdom level, respectively [8]. These methods perform quite well at oligonucleotide lengths as low as 4 bp. Yet another algorithm, RAIphy, calculates the log odds ratio between the observed and expected occurrence of each k-mer based on Markov assumptions for the k-mer probabilities [9]. This algorithm assigns a genomic fragment to a specific taxon based on a comparison between the two [9]. The possible weakness of these metagenomic methods is that they likely only use very short fragments as compared to the whole genome and thereby quite possibly skew their oligonucleotide frequency profiles. However, an analysis of the whole genome gives a certain and complete picture of these profiles.

Compared to our previous work in this area [1–3], the current improved algorithm scores k-mer significance based on a normalized scale of −1 to +1, which is used to calculate k-mer signatures so as not only to predict statistically significant and biologically relevant k-mers, but also to make the genomes of two given species comparable based on their k-mer signatures. Therefore, the goal of this study is to further develop a k-mer prediction method, which can be used to predict biologically significant k-mers and then to use these k-mers in species comparison and clustering.

The present method is novel in that it measures the Pearson correlation coefficient values of the normalized k-mer relevance scores (not just the k-mer's frequencies) between the whole genomes of a number of species and assigns them to clusters. While the underlying algorithm is similar, certain changes were made to differentiate statistically significant over- and underrepresented k-mers (see Materials and Methods). Furthermore, the k-mer prediction algorithm is now applied to a wider range of animal species as compared to plant species in the previous studies. This is because different genera of species provide sufficient diversity for cross-comparison, and the whole-genome sequences for these species were also available.

In this study, the whole-genome sequences of 22 *Anopheles* species, 30 *Drosophila* species, and six *Glossina* species from the NCBI database were downloaded (58 in total), analyzed, and compared with each other. We also included the whole-genome sequences of *Apis mellifera* and *Caenorhabditis briggsae* as outliers. Aside from the previously mentioned 58 species, the whole genomes of two *Aedes* and one *Culex* species were also downloaded and compared with the three Dipteran genera. These species were used as outlier species in order to measure how the genome of such unrelated species measures up to the species in the three genera under study. With a larger number of species involved, more general inferences can be made about k-mer content, as well as inferences about the phylogenetic aspects of two genera in relation to each other. These analyses have become possible because the whole-genome sequences of 110 fly species are available now, thus facilitating comparative studies with regard to gene content, genetic mechanisms, and genome structure [10].

*Anopheles* is a genus of mosquitoes, belonging to the family Culicidae and suborder Nematocera. *Anopheles* has 485 species, 100 of which can transmit malaria via the genus *Plasmodium*, and 41of the 100 species cause human malaria. 14 of the 22 *Anopheles* in this study are among those species, which cause malaria [11]. They are global in distribution and are studied mainly because of their epidemiological importance, having caused around 200 million deaths in 2013 [12]. The taxonomy of the subfamily Anophelinae is unstable. For example, a supposedly sister genus, *Bironella*, was classified by different groups either to be outside or within *Anopheles*. Morphological traits and DNA sequence data were studied to address the relationships between *Anopheles*, *Bironella*, and *Chagasia*, but were not able to produce stable results [13]. Therefore, the genome k-mer analysis of *Anopheles* species is a timely task.

The genus *Drosophila* (pomace, vinegar, or wine flies) [14] includes various multiple subgenera and clades and are widely distributed in the northern hemisphere. According to Throckmorton [15], the faunal disjunction of *Drosophila* species between the Old and New World occurred in five lineages (the Scaptodrosophilan, Sophophoran, virilis-repleta, immigrans-tripunctata, and the Hirtodrosophilan) [16]. The fruit fly species *Drosophila melanogaster* is probably the most well-known and widely studied insect in the world, due to its easy culturing, high reproductive rate and generation time, and small body size.

On a genomic level, *Anopheles* and *Drosophila* have several marked genomic differences. In general, anophelines have greater intron loss compared to drosophilids. They also have more genes, which are the result of gene fission and fusion events, affecting an average of 10.1% of all genes in the genomes of the 10 species with the most contiguous genome assemblies. Furthermore, codon usage is more uniform in anopheline genomes than in drosophilids [17].

Species of the genus *Glossina* (family Glossinidae, suborder Brachycera) or tsetse flies are characterized by difficult culturing, long generation times, and low reproductive rates. These fly species are studied due to their medical and economic importance of parasitism and their role as vectors of trypanosomes [18]. The genus consists of three subgenera, *Austenina*, *Nemorhina*, and *Glossina*, represented by the species, *G. fusca*, *G. palpalis*, and *G. morsitans*. Their 22 species were previously classified within five species complexes [19]. Extent tsetse flies are distributed in sub-Saharan Africa as well as the Saudi Arabian Peninsula [20].

With a novel method in hand, the goal of this study is to predict biologically important k-mers of lengths 7–9 bp in species identification by statistically examining all lexicographically possible k-mers for the 58 *Anopheles*, *Drosophila*, and *Glossina* species. K-mers, which are 8 bp long, correspond to the typical length of DNA which is recognized by transcription factors. Therefore, these k-mers are the length of typical core transcription factor binding sites [21, 22]. We also allowed for a ±1 bp wobble; this is why we chose a range of 7–9 bp. We achieve this by scoring the k-mers of the 58 species' motifome (defined as all lexicographically possible k-mers of a given length in the genome) based on their whole-genome sequences. Furthermore, with such a whole-genome k-mer signature (WGKS) available for each species (that is, the available scores of all k-mers in the genome of a given species), it will be possible to do an all-versus-all comparison for all the study species. This way, we could assign the species into different species clusters based on high correlations of the WGKS among members of the same cluster.

## 2. Materials and Methods

### 2.1. Sequence Data

The whole-genome sequences were downloaded from the NCBI Genome database (https://www.ncbi.nlm.nih.gov/genome/) for 22 *Anopheles* species, 30 *Drosophila* species, and six *Glossina* species. A genomic summary of these species including the names of the whole-genome sequences, the number of chromosomes/contigs/scaffolds present in their individual genomes, the size of their genomes, as well as the A/C/G/T % of their genomes is in Supplementary [Supplementary-material supplementary-material-1]. Two *Anopheles* species, *A. farauti* and *gambiae*, have two separate genomes in the database. One of their genomes was broken up into a large number of shorter fragments. Therefore, the genome with a smaller number of contigs was selected. The genome size and the ACGT% have been plotted for each of the 58 species in Supplemental Figures [Supplementary-material supplementary-material-1] and [Supplementary-material supplementary-material-1], respectively.

The 5′and 3′ UTR sequences for seven *Drosophila* species and the intron sequences for 12 *Drosophila* species were downloaded from the FlyBase database (ftp://ftp.flybase.net/genomes/). The 5′ and 3′ UTR sequence sets for *Anopheles gambiae* were also downloaded from the UTR database (http://utrdb.ba.itb.cnr.it/home/download) [23] as a comparison with the *Drosophila* species. Summary statistics for these species for 5′ and 3′ UTR regions as well as introns can also be seen in Supplementary [Supplementary-material supplementary-material-1].

The 28 mitochondrial genomes were downloaded from the NCBI database: https://www.ncbi.nlm.nih.gov/genome/browse#!/organelles. The genomes were aligned with the CLUSTALW2 software and trimmed so as to make the alignment less variable at the ends.

### 2.2. K-mer Scoring Algorithm

**Figure 1:**
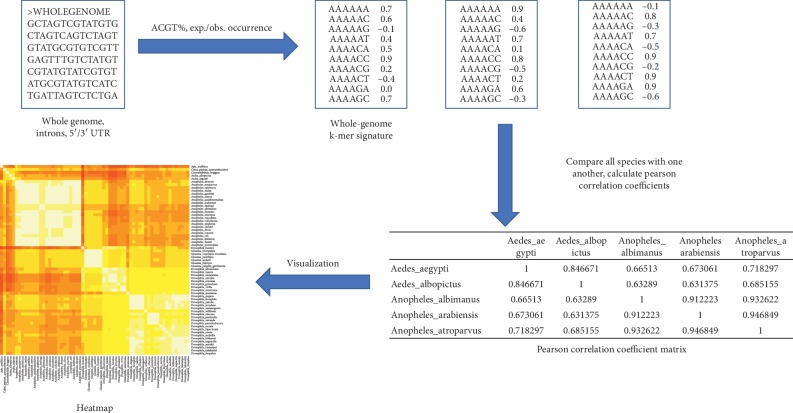
Flowchart depicting the algorithm. First, the whole-genome sequences or subgenomic region of interest for all species are analyzed, and the WGKS is produced. This is a list of all possible k-mers together with their normalized score values. These WGKSs are compared in an all-versus-all manner, using the Pearson correlation coefficient. This produces a CC matrix, which is then visualized in a heatmap, depicting species relationships.

The original k-mer scoring algorithm was described in the study of Lichtenberg et al. [21] and Cserhati et al. [1]. The algorithm is briefly described below; however, more details on the mathematical background for scoring the significance of a given k-mer can be found in the original publications. A flowchart showing the individual steps of the algorithm with inputs and outputs is shown in Figure 1.

The adapted algorithm used in the analysis is an enumeration algorithm, which counts the total occurrence of all possible k-mers of a given length *k* bp. A k-mer is viewed, for example, as the core section of a transcription factor binding site (TFBS) that different kinds of regulatory factors bind to, but it could also be a k-mer with any other kind of functional relevance. The k-mer sequence corresponds to a DNA surface that can specifically bind a regulatory protein. K-mers of lengths 7–9 bp were analyzed in this study (heptamers, octamers, and nonamers). For any length k, there are 4^k^ combinatorically possible k-mers, all making up the so-called motifome, as mentioned in the introduction. The longer the k-mer, the more specific its sequence is and the more well-defined its binding surface gets. The observed occurrence O of each k-mer was calculated for each possible k-mer.

For each genome, the background base pair distribution was also calculated in percent (A/C/G/T%). With this information, the probability of any given k-mer can be calculated based on Markov assumptions:

(1) $$\begin{array}{c} P\left(\text{expected}\right)=\overset{k}{\underset{i=1}{\prod }}p_{i}, \end{array}$$

where *p*_*i*_ is the percentage occurrence of the base at position *i* in the k-mer. These probabilities are multiplied together to get the expected probability of the given k-mer. The expected occurrence *E* of a given k-mer is equal to the length of the genome multiplied by the probability of the k-mer:

(2) $$\begin{array}{c} E=l_{\text{genome}}\cdot P\left(\text{expected}\right). \end{array}$$

In the previous works, a scoring algorithm was used to measure how much the actual occurrence O of a k-mer deviated from the expected occurrence E:

(3) $$\begin{array}{c} S_{\text{k}-\text{mer}}=\frac{\left|O-E\right|}{O}, \end{array}$$

where *S*_k-mer_ is the calculated score and *O* and *E* are the observed and expected occurrences of a given k-mer, respectively. The purpose of this score is to filter out meaningless k-mers that could simply occur by chance. If the expected and observed occurrences should be about the same, the score should be close to 0. However, if the observed occurrence of the k-mer is much greater than the expected occurrence, then the score is close to 1. If the expected occurrence *E* is much greater than O, then the score goes to infinity.

In this current study, equation (3) was modified to differentiate overrepresented and underrepresented k-mers. The new scoring equation is as follows:

(4) $$\begin{array}{c} S_{\text{k}-\text{mer}}=\frac{O-E}{O+E}. \end{array}$$

With this setup, there are three possible cases:

(5) $$\begin{array}{c} O\gg E:S_{\text{k}-\text{mer}}⟶1,\;\text{overrepresented k}-\text{mer,}\\ O\ll E:S_{\text{k}-\text{mer}}⟶-1,\;\text{underrepresented k}-\text{mer,}\\ O=E:S_{\text{k}-\text{mer}}\approx 0,\;\text{randomly occurring k}-\text{mer.} \end{array}$$

This way, all possible k-mers 7–9 bp long were scored for all 22 *Anopheles*, all 30 *Drosophila*, and all six *Glossina* species, as well as the two outlier species, *A. mellifera* and *C. briggsae*.

The input at this first stage of the algorithm is the whole-genome sequences of all species in the study. The whole-genome sequence is used to calculate the expected and observed occurrences of all 4^k^ k-mers. The output is the WGKS for all species, a two-column list including the k-mers and their scores.

The list of k-mers and their occurrence and score values are all available in the Supplemental material online. The python script that performs the analysis is publicly available on github at https://github.com/csmatyi/motif_analysis for interested users.

### 2.3. Calculation of Correlation between Any Two Species Based on K-mer Scores and Heatmap

The input of this stage of the algorithm is the WGKSs for all species in the study from the previous step. The output is a symmetric matrix of Pearson correlation coefficients (CC) for all species pairs showing how well their WGKSs correlate with one another.

The Pearson correlation coefficient between any two species was calculated based on their k-mer scores for any given k-mer length (here 7–9 bp). For any two species under consideration, all possible k-mers of length *k* were sorted lexicographically from *A*_*k*_ to *T*_*k*_ (*k* = 7–9). If any k-mer was missing from either species, then it was omitted. The correlation coefficient was calculated based on the scores for each k-mer present in both species. These correlation coefficient values were depicted in a heatmap, one for each k-mer length from 7 to 9 bp.

We have three genera (*Drosophila*, *Anopheles*, and *Glossina*) in this study. The group is defined as all species in a specific genus and the nongroup is defined as all the remaining species. To compare the statistical significance of the CC values between any two species within a group vs. all CC values of all correlations between any one species in a group and any one species in a nongroup, we performed the Welch's *t*-test (unequal variance) for each comparison.

### 2.4. Creation of Plots

In the last step of the algorithm, the symmetric CC matrix is transformed into a heatmap to depict the relationship between all species in the study. Barplots, boxplots, and heatmaps were generated using the barplot, boxplot, and heatmap functions in R, version 3.4.3. Phylogenetic trees for the three insect genera were created in R using the library phangorn, using the commands upgma. The CC values for octamers were subtracted from 1 to get distance values, which were then used in the upgma command. Venn diagrams were created with the online software at http://bioinformatics.psb.ugent.be/webtools/Venn/.

### 2.5. Phylogenetic Trees

Phylogenetic trees were created for all three insect genera using the phangorn library in R. The distance metric was 1−CC for all species pairs. Trees were created using the UPGMA, WPGMA, and NJ methods, using the upgma, wpgma, and nj commands.

### 2.6. Taxonomical Comparisons

The classification of *Drosophila* species was matched to data (Genus/Subgenus/Group/Complex) in the TaxoDros Database at http://www.taxodros.uzh.ch.

### 2.7. Acquisition of Tandem Repeat Sequences

Tandem repeat sequences were retrieved from the Tandem Repeats Database at https://tandem.bu.edu/cgi-bin/trdb/trdb.exe [24]. Repeats of length 8 with 0 mismatches were selected for *D. mojavensis*.

### 2.8. Matching Biologically Relevant Genome K-mers against Position Weight Matrixes in the JASPAR Database

Position weight matrixes (PWM) for 140 transcription factor binding sites (TFBS) in *D. melanogaster* were downloaded from the JASPAR website [25] at http://jaspar.genereg.net. For all 30 *Drosophila* species, all biologically relevant candidate octamer k-mers were matched against all of 140 of these PWMs in a sliding window-like manner (since not all octamers were as long as these PWMs). A cutoff of 80% sequence similarity was used to call a match between a given k-mer and a JASPAR k-mer (which is the default used by the JASPAR database). Each JASPAR database hit is listed next to each k-mer in Supplementary [Supplementary-material supplementary-material-1].

Putative biologically relevant genome k-mers were determined for a given species by calculating the mean score and standard deviation for each species and using the mean ± 2SD value as a cutoff. All k-mers with a score value above this limit were predicted as biologically relevant. This is because in the normal distribution, 5% of all values lie above 1.96 *z*-score limit. This cutoff was also used in the k-mer prediction study of modern and archaic humans [3].

## 3. Results and Discussion

### 3.1. Whole-Genome Sequence Analysis

For each species, the whole-genome motifome was enumerated and scored for k-mers of lengths 7–9 bp. Then, the whole-genome k-mer content was compared in an all-versus-all pairwise fashion, to determine correlation coefficients of 1,953 comparisons in total (including comparisons between the same species). These values are available in Supplementary [Supplementary-material supplementary-material-1] for k-mers of lengths 7–9 bp.

The CC value represents how similar the WGKSs are between two species. Similar WGKSs of two species in turn reflect how similar the genomes are between these two species. Obviously, a more similar pair of species will contain more similar distribution of k-mers throughout their genomes and thus have a higher CC value. This is because on a macroscopic level, the genomes of similar species have not had enough time to diverge and accumulate too many mutational differences. Conversely, the distribution of k-mers in the genomes of dissimilar species is different, and thus, their WGKSs are also different. Therefore, they also contain k-mers (for example, transcription factor binding sites) with different functions. For example, in a study of *D. melanogaster*, *D. simulans*, *D. erecta*, and *D. yakuba*, 5% of functional Zeste transcription factor binding sites were gained and/or lost compared to the other lineages [26].

**Figure 2:**
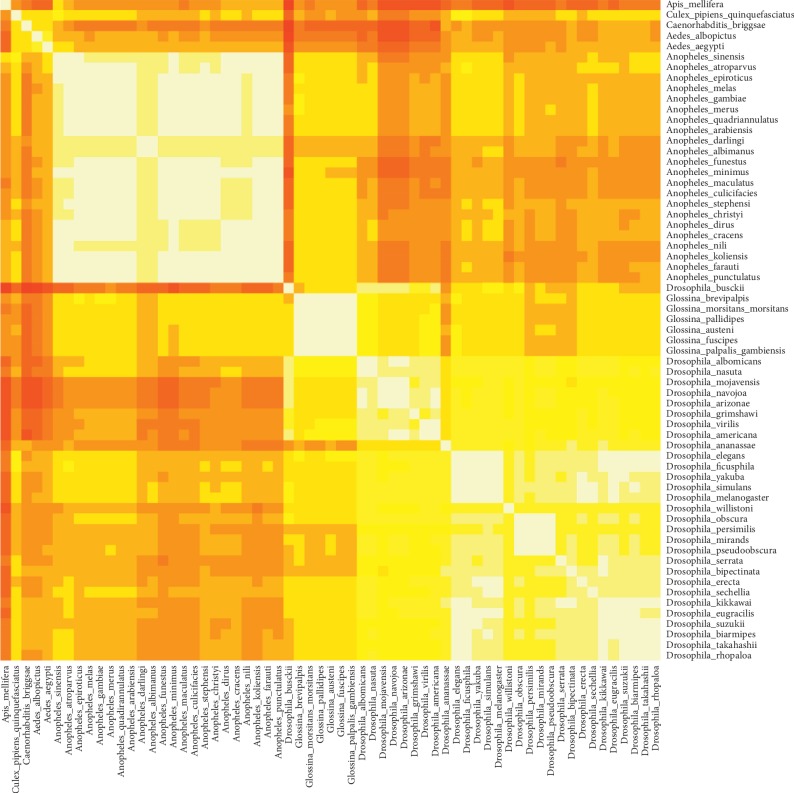
Heatmap depicting CC values calculated in an all-versus-all pairwise manner between the 63 species included in the analysis based on the whole-genome k-mer signature for octamers. Colors closer to yellow or white indicate higher CC values, while those closer to red indicate lower CC values. The range of the CC values in this matrix is from 0.259 to 1.0.

The CC matrixes for the 63 species are depicted in the heatmaps in Figure 2 based on octamers. In the heatmap, a lighter, yellower color denotes a higher CC value, closer to 1, denoting species whose WGKS is similar to one another. Darker, redder colors denote CC values closer to 0, denoting species pairs with an unrelated WGKS. What is very clear is that the three genera, *Anopheles*, *Drosophila*, and *Glossina* clearly separate from one another quite well and also from the two outliers.

#### 3.1.1. *Drosophila*

**Figure 3:**
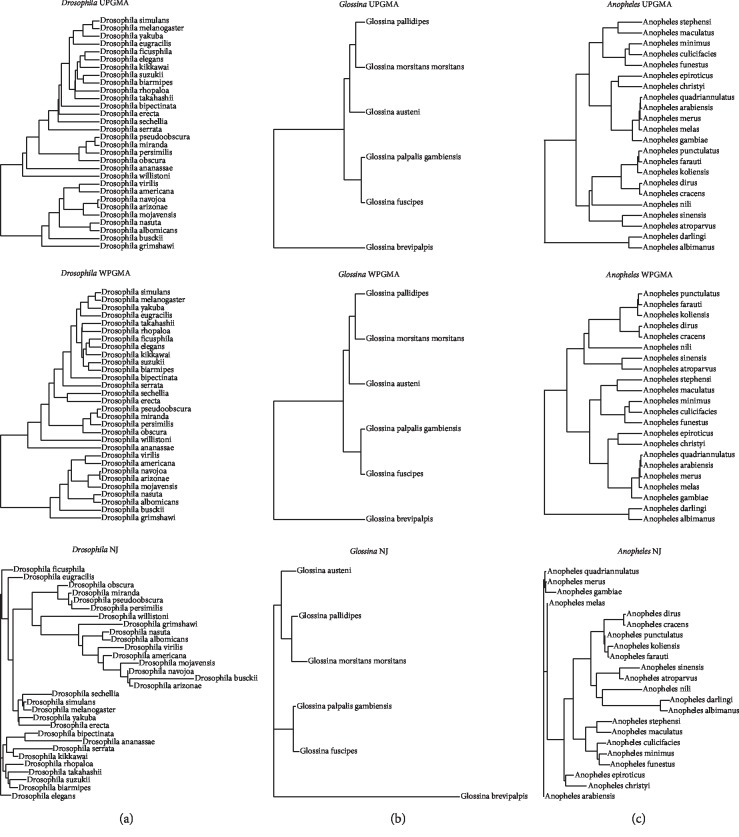
UPGMA, WPGMA, and NJ trees for 1−CC values for all species pairs from each of the three genera: (a) *Drosophila*, (b) *Glossina*, and (c) *Anopheles*.

Within the *Drosophila* cluster, a smaller subgroup can be seen including eight species: *D. albomicans*, *D. americana*, *D. arizonae*, *D. grimshawi*, *D. mojavensis*, *D. nasuta*, *D. navajoa*, and *D. virilis*. These species represent a separate monophylogenetic group within the genus *Drosophila* and correspond to the subgenus *Drosophila*. All of the other species belong to the subgenus *Sophophora*. Within *Sophophora*, four species can be seen which themselves form a small, compact group: *D. miranda*, *D. obscura*, *D. persimilis*, and *D. pseudoobscura*. These four species belong to the obscura species group within *Sophophora*. The phylogenetic tree for the genus *Drosophila* can be seen in Figure 3(a). Trees using the UPGMA, WPGMA, and NJ methods were drawn as described in the Materials and Methods section. The *Drosophila* and *Sophophora* separate well from one another. The outlier species *D. ananassae*, *busckii*, and *willistoni* also separate well from all of the other species.

This algorithm was used not only for measuring species similarity based on correlation of k-mer content, but also for predicting biologically relevant genome k-mers in all three Dipteran genera, as described in the Materials and Methods section. A list of all putative biologically relevant octamers is provided in Supplementary [Supplementary-material supplementary-material-1]. A summary of these predicted k-mers can be seen in Table 1.

It was found that shorter k-mers are more conserved because it is harder to conserve longer stretches of DNA. However, the shorter the k-mer, the less possible number of k-mers can be studied. Shortening k-mers loses information and precision because longer k-mers increase the k-mer signature, making the calculation of the CC value more precise. For octamers, the mean CC value was 0.857 (std. dev. 0.07). A *p* value of unequal variance of 2.3 × 10^−247^ was calculated for CC values within *Drosophila* and CC values between *Drosophila* and non-*Drosophila* species. A Cohen's *d*-value of 3.18 (CI of 3.03–3.32, 95% confidence level) was calculated, which is very high.

#### 3.1.2. *Drosophila* Species with High Repeat Content in Their Genomes

Another species of *Drosophila* (*D. busckii*) is seemingly misplaced on the heatmap, between *Anopheles* and *Glossina*, away from the other *Drosophila* species. This species has the lowest average CC value compared to all other *Drosophila* species (0.753, octamers, Supplementary [Supplementary-material supplementary-material-1]). However, when the CC value of *D. busckii* was compared to that of the six members of the genus *Glossina*, the average CC value was 0.699 (looking at octamers). When comparing CC values between *D. busckii* and *Glossina* versus *D. busckii* and all other *Drosophila*, the *p* value was 0.006. When comparing *D. busckii* only with the eight members of this small monophyletic group within *Drosophila*, an average CC of 0.891 can be calculated, with a *p* value of 4.7 × 10^−9^, when comparing CC values between *D. busckii* and *Glossina* versus *D. busckii* and these eight *Drosophila* species. The TaxoDros Database also classifies *D. busckii* in its own separate species group (the busckii species group, which is part of the *Dorsilopha* subgenus). It is not exactly certain why *D. busckii* clusters the way it does. Zhou and Bachtrog [27] have observed that 60% of the neo-Y-linked genes have become nonfunctional in *D. busckii*. Therefore, it is possible that due to this, the regulatory motifs in their promoter regions have also undergone differential mutations, thereby altering the k-mer content of this species.

*D. ananassae*, a species belonging to the *Drosophila* subgenus, shows lower resemblance to the members of the *Sophophora* subgenus. This can be seen well in Figure 2. *D. ananassae* is the species with the next lowest average CC value (0.800, octamers, Supplementary [Supplementary-material supplementary-material-1]) to all other *Drosophila* species. This could be due to the fact that its genome has the highest percent content of repetitive elements (24.93%), followed by *D. willistoni* (15.57%), also with the fifth lowest average CC value among the drosophilids (0.832, octamers, Supplementary [Supplementary-material supplementary-material-1]). A high repetitive element content in a species' genome means that the observed occurrence of many k-mers will be increased, thereby skewing the score for that specific k-mer. This in turn will also decrease the CC value between the given species and other species which do not have a high repetitive element content. These two species also have the highest number of pseudotransfer (t)RNA genes (*D. ananassae*—165/472; *D. willistoni*—164/484). Indeed, of the 81 of the 98 reverse complement k-mers of *D. ananassae* with a minimum score of 0.8 and a minimum occurrence of 10,000, only 6–14 were also found in any of the other *Drosophila* species, also with a minimum score of 0.8 and a minimum occurrence of 10,000. For *D. willistoni*, of the 44 of the top 46 such abundant high-scoring reverse complement k-mer, only 8–22 were found also to be high-scoring in the genome of any other *Drosophila* species, except for the genome of *D. mojavensis*, which had 30 such high-scoring abundant reverse complement k-mers (see Supplementary [Supplementary-material supplementary-material-1] for lists). This indicates that these abundant, high-scoring repetitive k-mers might be the reason for skewing the CC values between *D. ananassae* and *D. willistoni* and all other species.

*D. mojavensis* is another species that clusters well with other species from the subgenus *Drosophila*, but still had the third lowest CC value (0.823, octamers, Supplementary [Supplementary-material supplementary-material-1]). 592 octamer k-mers for this species without any mismatches were selected from the Tandem Repeats Database (TRDB) [24]. These k-mers were filtered if they had a score less than 0.333. According to equation (4) in the Materials and Methods section, this corresponded to a k-mer which occurred twice as many times as its expected occurrence and therefore serves as a good cutoff CC value to gauge functional biological relevance. 245 of these 592 k-mers (41.4%) from the WGKS of *D. mojavensis* had a score higher than or equal to 0.333. *D. mojavensis* had 86 abundant, high-scoring reverse compliment k-mers (see filtering criteria in the previous paragraph). Five other *Drosophila* species had at least half as many (43) such specific k-mers, including *D. busckii*, which as seen before had the lowest mean CC value with all other *Drosophila* species (Supplementary [Supplementary-material supplementary-material-1]). *D. mojavensis* and *D. busckii* have a CC value of 0.88 (octamer level), which is above both the mean and the median within *Drosophila*. This also indicates that the high repeat k-mer content of this species may be skewing its CC values with other *Drosophila* species as well.

#### 3.1.3. *Glossina*

The *Anopheles* and *Glossina* clusters are much more compact than *Drosophila*. The mean CC between all six *Glossina* species was 0.978 with a std. dev. of 0.02 (looking at octamers, see Table 2), whereas the average CC between *Glossina* and non-*Glossina* species was 0.761 with a std. dev. of 0.143 (Table 2). The *p* value is 1.5 × 10^−18^ comparing within-*Glossina* CC values versus CC values between *Glossina* and non-*Glossina* species. A Cohen's *d*-value of 8.48 (CI of 7.79–9.18, 95% confidence level) was calculated, which is very high.

Supplementary Figures [Supplementary-material supplementary-material-1] and [Supplementary-material supplementary-material-1] also show that both the genome size (315–380 Mbp) and the ACGT% are also relatively invariable compared to the other two Dipteran families. This might be due to the relatively small number of species examined and also the close relationship of the six species examined. On the heatmap, *G. brevipalpis* is correctly classified into its own group, corresponding to the subgenus *Austenina*. *G. morsitans morsitans* and *G. pallipides* on the heatmap correctly cluster together and belong to the subgenus *Glossina*. *G. fuscipes* and *G. palpalis gambiensis* also cluster together on the heatmap as part of the subgenus *Nemorhina*. One species, *G. austeni*, however clusters together with the palpalis group, whereas according to NCBI taxonomy it belongs to the subgenus *Glossina*. These species relationships are also mirrored in the phylogenetic tree in Figure 3(b). All three phylogenetic algorithms produce the same species relationships as described previously.

#### 3.1.4. *Anopheles*

The mean CC value calculated for octamers between *Anopheles* species was 0.948 (std. dev. 0.023). A *p* value of 0.0 was calculated for CC values within *Drosophila* and CC values between *Anopheles* and non-*Anopheles* species (meaning that the *p* value was too low that the neglog value cannot be displayed). A Cohen's *d*-value of 3.18 (CI of 5.03–5.47, 95% confidence level) was calculated, which is very high.

Hao et al. [28] performed a phylogenetic analysis based on 13 conserved mitochondrial protein-coding genes from 50 mosquito species. Based on their phylogenetic tree, the species from the *Anopheles* cluster as well as the *Aedes* and *C. quinquefasciatus* clustered similarly in the Hao et al.'s study and also in the present study. For example, *A. darlingi* is located on a separate major branch in the Hao study, and in the present study, this species as well as *A. albimanus* grouped together within the *Anopheles* cluster, within the subgenus *Nyssorhynchus* (within the genus *Anopheles*, subfamily Anophelinae). These two species had a CC value of 0.989 (looking at octamers, see Supplementary [Supplementary-material supplementary-material-1]), whereas the average CC value between these two species and the rest of the *Anopheles* cluster is 0.955 (see Supplementary [Supplementary-material supplementary-material-1]). In both the heatmaps and Figure 6 of Hao et al. [28], *A. arabiensis*, *gambiae*, *melus*, and *merus* cluster together, corresponding to the *gambiae* species complex of the subgenus *Cellia* of the genus *Anopheles*. In the heatmaps, *A. farauti* and *koliensis* cluster together, and in the phylogenetic tree of Hao et al.'s study, these two species also cluster on the same major branch. Also, the species *A. cracens* and *dirus* cluster together closely in both the heatmap and the phylogenetic tree. In the Hao et al. study [28] and also on the heatmap, the species *A. sinensis* and *atroparvus* also cluster together. These two species are members of the *Anopheles* subgenus of the genus *Anopheles*.

In another study by Freitas et al. [29], cytochrome oxidase subunits I and II (COI and COII) as well as the 5.8 S ribosomal subunit were analyzed to study the phylogenetic relationships between 47 *Anopheles* species. In their study as well as the present one, *A. farauti*, *koliensis* and *punctulatus* all clustered together, which are part of the *Anopheles punctulatus* group, which are major malaria vectors in the Southwest Pacific [30]. *A. arabiensis*, *gambiae*, *melus*, and *merus* also clustered closely in both studies, just as they did in the Hao et al.'s study [28]. However, whereas in the heatmaps *A. dirus* and *stephensi* clustered together, they were located on separate branches of the phylogenetic trees in both the Hao and the Freitas studies.

This difference might be due to both the Hao and Freitas studies having analyzed only the mitochondrial genome as opposed to the whole-genome studies in this paper. Nevertheless, these close clusterings between all three studies are remarkable in that very similar results were derived from analyzing a handful of mitochondrial proteins as well as from a global sequence analysis of the whole genome. The phylogenetic tree for *Anopheles* can be seen in Figure 3(c). The UPGMA and WPGMA trees look similar to one another, whereas the NJ tree looks somewhat different.

#### 3.1.5. Species Relationships Based on Alignment of Mitochondrial DNA

**Figure 4:**
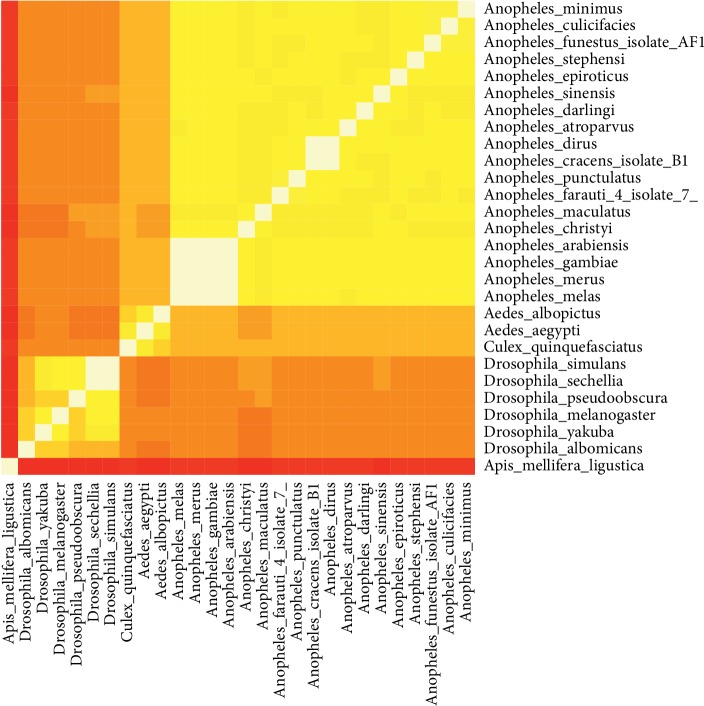
Heatmap depicting similarity of the mitochondrial genomes across 28 species. Lower similarity values are shown in darker, redder colors, closer to 0% similarity, whereas higher similarity values, closer to 100%, are shown in brighter, yellow/white colors. The range of similarity values is between 0 and 100%.

The mitochondrial whole-genome sequence for 29 species (19 *Anopheles*, 6 *Drosophila*, 2 *Aedes*, 1 *Culex*, and 1 *Apis*) was downloaded from the NCBI database. These sequences were aligned, and then, the percent identity was calculated for each possible pairwise species pair. These identity values are depicted in Figure 4 and are also available in Supplementary [Supplementary-material supplementary-material-1].

Figure 4 depicts species from the genera *Anopheles* and *Drosophila* segregating into two well-defined groups. The *p* value for *Anopheles* is 6.2 × 10^−81^, whereas for *Drosophila* it is 8.9 × 10^−9^. The two *Aedes* species group together, along with *Culex quinquefasciatus*. The outlier species, *Apis mellifera*, groups well away from all of the other species.

Within the genus *Drosophila*, only *D. albomicans* belongs to the subgenus *Sophophora*, whereas the other five species belong to the subgenus *Drosophila*, supporting previous results coming from the analysis of the WGKS.

Within the genus *Anopheles*, four species, *A. arabiensis*, *gambiae*, *melas*, and *merus* are very similar according to both their mtDNA, which reinforces the previous results from the analysis of the WGKS. Another group of species which cluster tightly together are *A. farauti*, *punctulatus*, *cracens*, and *dirus*. These four species also cluster tightly on Figure 2. Five other species, *A. culicifacies*, *epiroticus*, *funestus*, *minimus*, and *stephensi*. These species do not cluster together in Figure 2. This difference could simply be due to the fact that the k-mer profiles of the 28 species in question here reflect the k-mer distribution of the mtDNA only, and not that of the whole entire genome.

#### 3.1.6. Classification of New Species Based on WGKS

Since the taxonomy of many insect groups is in flux, it was interesting to see how several species from different genera were classified according to this algorithm. The WGKS of two *Aedes* species, *A. aegypti* and *A. albopictus*, and of *Culex quinquefasciatus* (all three being mosquito species) were analyzed and compared to species of *Anopheles*, to see if they form a separate group, or if they possibly form a monophyletic group together with *Anopheles*. Whole-genome sequences for species in the genera *Bironella* or *Chagasia*, the two closest genera to *Anopheles* in the subfamily Anophelinae were not available at NCBI. In Figure 2, all three species separate from the genus *Anopheles*. The two *Aedes* species have an average CC of 0.651 with *Anopheles*, whereas they have a CC of 0.847 between themselves (when looking at octamers, see Supplementary [Supplementary-material supplementary-material-1]). When comparing the CC values between *Aedes* and *Anopheles* to the CC values within the genus *Anopheles* itself (Supplementary [Supplementary-material supplementary-material-1]), a *p* value of 9.1 × 10^−54^ can be calculated. Thus, it can be concluded that *Aedes* form a group separate from *Anopheles*. When comparing *C. quinquefasciatus* to *Anopheles*, the mean CC value is 0.706. This is significantly different than the mean CC of *Anopheles* species among themselves (0.948, see Table 2, also looking at octamers). The *p* value between these two sets of CC values is 5.7 × 10^−23^. Therefore, it can be inferred that *C. quinquefasciatus* is also separate from the genus *Anopheles*.

This shows that the present method is useful in classifying as of yet unknown organisms for which only the whole-genome sequence is available. The utility of comparing WGKS is greater than methods which analyze only groups of genes, which make up only a fraction of the entire genome sequence. Phylogenies based on different genes often conflict with each other [6].

#### 3.1.7. Divergence and Similarities between Genera

**Figure 5:**
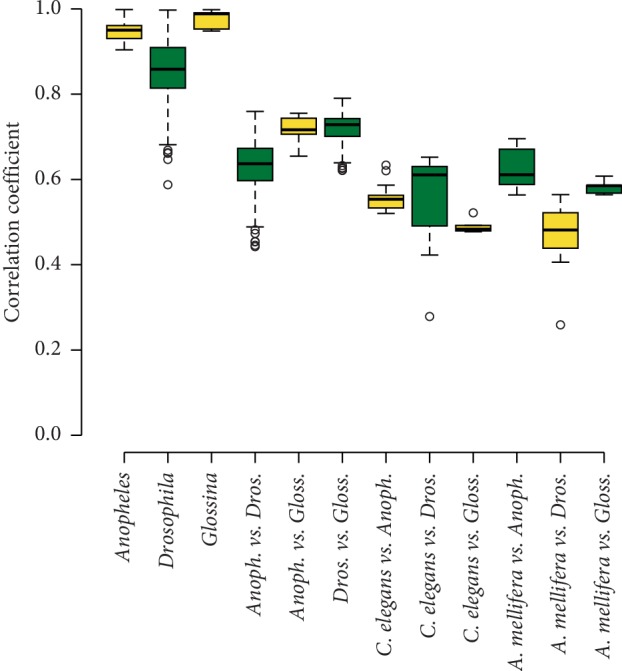
Pearson correlation coefficient (CC) values between species of Anopheles, Drosophila, and *Glossina* as well as the two control species, A. *mellifera* and *C. elegans* for *octamers*. The first three columns represent CC values between all pairs of species within each genera of Anopheles, Drosophila, and *Glossina*, respectively; columns 4–6 represent comparisons across the species from three genera, 7–9 represent comparison between C. *elegans* and the three genera, while 10–12 represent comparison between A. *mellifera* and the three genera.

In order to measure the divergence of the two genera from each other, boxplots were created comparing the range of CC values within the genera *Anopheles*, *Drosophila*, and *Glossina* as well as between the three genera themselves, as well as between *C. briggsae* and the three insect genera individually, and also between *A. mellifera* and the three insect genera individually. This was done for k-mers of size 7–9 bp, and the boxplots can be seen in Figure 5 (octamers) and Supplementary Figures [Supplementary-material supplementary-material-1] and [Supplementary-material supplementary-material-1] (heptamers and nonamers). The minimum, median, average, and maximum CC value for each of the seven comparisons as well as their standard deviations can be seen in Table 2.

The mean CC values within the three genera are much higher than for all other comparisons (e.g., 0.955 within *Anopheles* and 0.869 within *Drosophila* for heptamers, Table 2), whether they are between the two genera *Anopheles* and *Drosophila* or between either one of the two outlier species and either one of these two genera. This trend is consistent for all k-mer lengths. The minimum, mean, median, and maximum CC values decrease with increasing motif length, but this is due to the fact that as the motif length increases, the number of possible k-mers also increases proportionally, and therefore, CC values also tend to decrease. These tendencies all illustrate the clear genomic content differences between the genera *Drosophila*, *Anopheles*, and *Glossina*.

**Figure 6:**
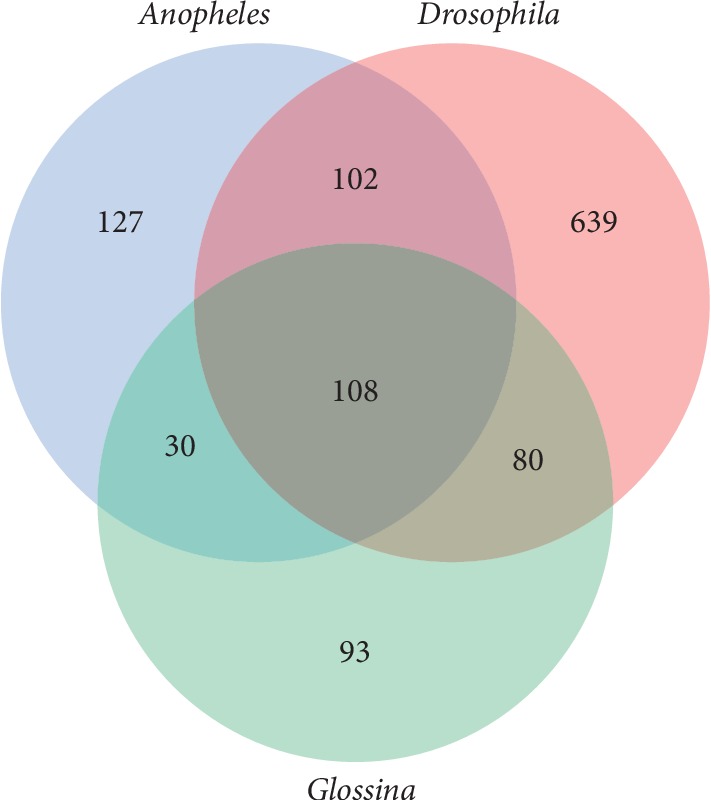
Common nonrepetitive (nondimer and nontrimer) octamer content between 11 *Anopheles*, 15 *Drosophila*, and 5 *Glossina* species. Each included octamer had a minimum score of 0.5.

It was also interesting to see which nonrepetitive (i.e., k-mers which do not consist of dimer or trimer repeats) genome k-mers were the most common between *Anopheles, Drosophila*, and *Glossina* for k-mers of lengths 7–9 bp. For this, all k-mers with a score of at least 0.5 (such k-mers occur three times more frequently than expected) and which occurred in at least half of all species in a given genus were selected (at least 11 *Anopheles* species and at least 15 *Drosophila* species, but at least 5 species in *Glossina*). These k-mers are listed in Supplementary [Supplementary-material supplementary-material-1] for lengths 7–9 bp. Common k-mers between all three genera are also listed in Supplementary [Supplementary-material supplementary-material-1] and are visualized in Figure 6 (octamers) and Supplementary Figures [Supplementary-material supplementary-material-1] and [Supplementary-material supplementary-material-1] (heptamers and nonamers).

### 3.2. Analysis of 5′ and 3′ UTRs

Besides the whole genome, k-mer analysis was done for 5′ and 3′ UTRs for seven *Drosophila* species (D. *ananassae*, *erecta*, *grimshawi*, *melanogaster*, *mojavensis*, *pseudoobscura*, and *simulans*) and also *Anopheles gambiae* as an outlier species which was compared to these *Drosophila* species. Besides the WGKS, a species' 5PKS, 3PKS, and also IKS (5′ prime k-mer signature, 3′ k-mer signature, and intron k-mer signature) can also be defined. Sequence statistics for the selected *Drosophila* species and *A. gambiae* are available in Supplementary [Supplementary-material supplementary-material-1]. However, since 5′ and 3′ UTR sequences were not available for many species besides *Drosophila*, we could only do a restricted analysis, instead of analyzing species relationships on a heatmap as in Figure 2.

**Figure 7:**
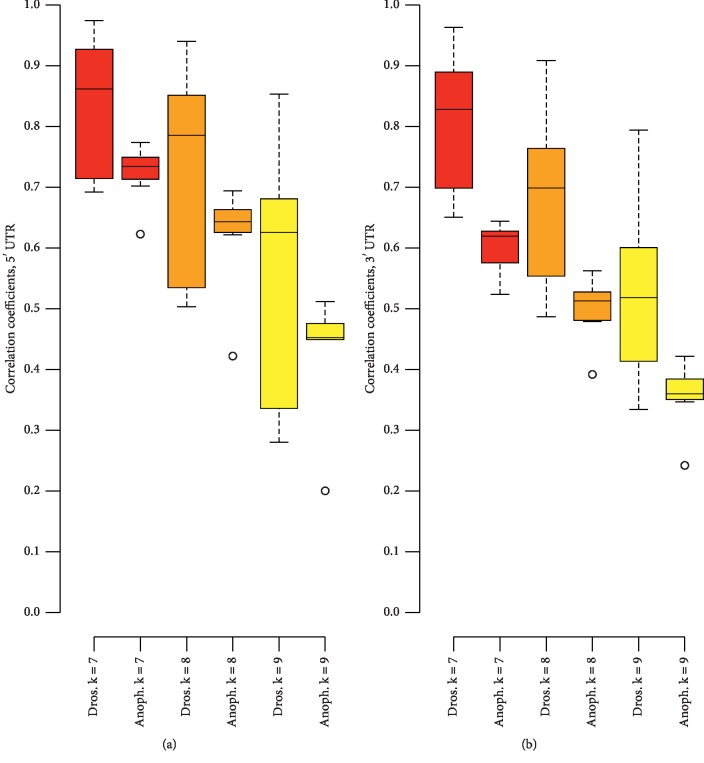
Comparison of the similarity in the 5′ and 3′ UTRs with the genus of *Drosophila* and between the species of *Drosophila* and *A. gambiae*, using k-mers of lengths 7–9 bp: (a) 5′ UTR and (b) 3′ UTR. Yellow bars represent comparisons among *Drosophila* species, and green bars represent comparison between *Drosophila* species and *A. gambiae*.

Figures 7(a) and 7(b) depict the CC ranges in boxplots for both within the genus *Drosophila* and between *A. gambiae* and the genus *Drosophila* for k-mer lengths 7–9 bp for 5′ and 3′ UTRs, respectively. Both figures show that the CC range for comparisons between *A. gambiae* and *Drosophila* is much lower than that for within *Drosophila* itself. This difference between the two genera is more pronounced in 3′ UTRs as compared to 5′ UTRs. The CC values are present in a matrix for both 5′ and 3′ UTRs in Supplementary Files [Supplementary-material supplementary-material-1] and [Supplementary-material supplementary-material-1], respectively.

**Figure 8:**
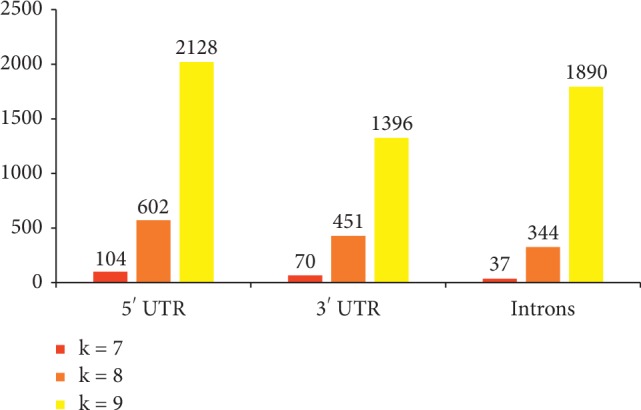
Number of common k-mers of lengths 7–9 bp for all seven *Drosophila* species for 5′ and 3′ UTRs and introns.

Summary statistics for CC values within *Drosophila* and between *A. gambiae* and *Drosophila* can be seen in Table 3. The *p* values for 5′ and 3′ UTRs for k-mer lengths 7–9 bp are all statistically significant at the 5% level. This reflects that the same kind of genetic difference between the two genera is also present in the 5′ and 3′ UTR regions.  Figure 8 shows the number of 5′ UTR nonrepetitive k-mers which are common to all seven *Drosophila* species, 104, 602, and 2128 for motif lengths 7–9 bp. For 3′ UTRs, there are 70, 451, and 1396 motifs of lengths 7–9 bp. This is reflective of the lower overall CC range for 3′ UTR k-mers than 5′ UTR k-mers seen earlier (Figures 7(a) and 7(b)). The number of common k-mers increases in a roughly proportionate manner as the length of the k-mer increases, due to increasing k-mer space (e.g., there are more possible nonamers than octamers). These common k-mers are listed in Supplementary Files [Supplementary-material supplementary-material-1] and [Supplementary-material supplementary-material-1] for 5′ and 3′ UTRs.

### 3.3. Analysis of Introns

The intron regions of twelve *Drosophila* species (*ananassae*, *erecta*, *grimshawi*, *melanogaster*, *mojavensis*, *persimilis*, *pseudoobscura*, *sechellia*, *simulans*, *virilis*, *willistoni*, and *yakuba*) were analyzed in a way similar to the whole genome as well as the 5′ and 3′ UTR regions. Intron sequences were available for only *Drosophila*; therefore, we could not perform any species comparisons between this genus and *Anopheles* or *Glossina*.

**Figure 9:**
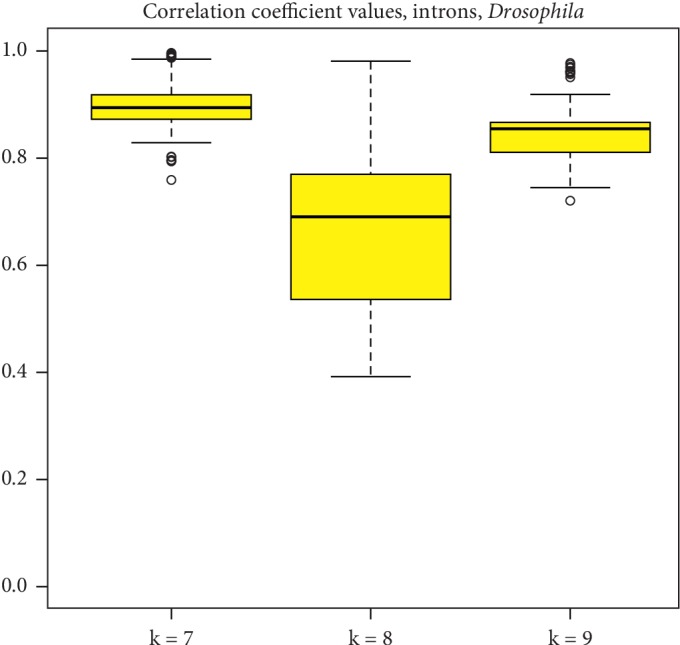
Pearson correlation coefficient values range from all-versus-all comparison of twelve *Drosophila* species for k-mer lengths 7–9 bp which have data from the intron regions.

Figure 9 depicts the range of CC values for k-mer lengths 7–9 bp for these twelve species. Summary statistics for all k-mer lengths are available in Table 3. The number of common k-mers to all twelve species is depicted in Figure 8 (37, 344, and 1890 for motif lengths 7–9 bp) and is listed in Supplementary [Supplementary-material supplementary-material-1], where the CC matrix is also available for k-mer lengths 7–9 bp.

As with the 5′ and 3′ UTR regions, the number of common k-mers also increases with increasing k-mer length, from 7 to 9 bp. The number of common intron k-mers is also less than the number of common 3′ UTR k-mers, which in turn is less than the number of common 5′ UTR k-mers (for heptamers and octamers), but not for nonamers (see Figure 8). This indicates that, for these two k-mer lengths, as the size of the sequence regions decreases, the number of common k-mers increases.

## 4. Conclusion

The motif prediction algorithm presented in previous works has been refined, expanded, and applied to a lot larger selection of species, allowing broader inferences to be made from the analysis. Furthermore, by defining the WGKS of yet unknown species, they can be classified into existing taxonomical categories. This algorithm is one more tool with which to characterize and classify new species, as in the case of *A. aegypti* and *albopictus* and *C. quinquefasciatus*. The WGKS, but also motif signatures from other subgenomic regions, can be useful in separating species into individual genera, sharply separated from one another. We believe that this algorithm can be put to use to not only predict biologically relevant whole-genome and subgenomic motifs, but also cluster species into taxonomic groups based on similarities and differences among their motif signatures. This algorithm has only been used to analyze insect species, but could also be applied to compare species from other phyla.

## Figures and Tables

**Table 1:** Number of statistically significant genome k-mers and minimum score for all species.

Species	No. of significant k-mers	Min. score	No. of hits in JASPAR database
Anopheles_albimanus	1646	0.383	NA
Anopheles_arabiensis	1629	0.349	NA
Anopheles_atroparvus	1425	0.346	NA
Anopheles_christyi	1366	0.414	NA
Anopheles_cracens	1523	0.433	NA
Anopheles_culicifacies	1440	0.371	NA
Anopheles_darlingi	1646	0.413	NA
Anopheles_dirus	1648	0.387	NA
Anopheles_epiroticus	1562	0.375	NA
Anopheles_farauti	1397	0.435	NA
Anopheles_funestus	1579	0.340	NA
Anopheles_gambiae	1509	0.394	NA
Anopheles_koliensis	1309	0.461	NA
Anopheles_maculatus	1613	0.377	NA
Anopheles_melas	1551	0.379	NA
Anopheles_merus	1755	0.281	NA
Anopheles_minimus	1406	0.379	NA
Anopheles_nili	1206	0.427	NA
Anopheles_punctulatus	1276	0.456	NA
Anopheles_quadriannulatus	1771	0.270	NA
Anopheles_sinensis	1381	0.419	NA
Anopheles_stephensi	1666	0.369	NA
Drosophila_albomicans	2279	0.428	23
Drosophila_americana	2209	0.428	22
Drosophila_ananassae	2067	0.481	22
Drosophila_arizonae	2293	0.405	19
Drosophila_biarmipes	1899	0.475	19
Drosophila_bipectinata	1934	0.449	15
Drosophila_busckii	2406	0.442	25
Drosophila_elegans	1768	0.519	19
Drosophila_erecta	2047	0.470	17
Drosophila_eugracilis	1838	0.424	21
Drosophila_ficusphila	1591	0.435	19
Drosophila_grimshawi	2377	0.465	16
Drosophila_kikkawai	1834	0.468	16
Drosophila_melanogaster	1805	0.472	20
Drosophila_miranda	1973	0.429	28
Drosophila_mojavensis	2435	0.435	17
Drosophila_nasuta	1981	0.468	15
Drosophila_navojoa	2239	0.508	19
Drosophila_obscura	2029	0.500	22
Drosophila_persimilis	2111	0.423	24
Drosophila_pseudoobscura	2046	0.393	26
Drosophila_rhopaloa	1757	0.427	17
Drosophila_sechellia	1883	0.456	20
Drosophila_serrata	1820	0.410	15
Drosophila_simulans	1758	0.496	21
Drosophila_suzukii	1937	0.442	21
Drosophila_takahashi	1834	0.364	20
Drosophila_virilis	2415	0.475	23
Drosophila_willistoni	2223	0.425	21
Drosophila_yakuba	1843	0.410	19
Glossina_austeni	1741	0.367	NA
Glossina_brevipalpis	1973	0.360	NA
Glossina_fuscipes	1787	0.370	NA
Glossina_pallidipes	1732	0.373	NA
Glossina_palpalis_gambiensis	1810	0.342	NA
Glossina_morsitans_morsitans	1735	0.377	NA

**Table 2:** CC statistics for k-mers of lengths 7–9 bp for different combinations of the genera under study.

Group comparison	Min	Median	Mean	Max	Std. dev.	No. of comparisons
Heptamers						
*Anopheles*	0.913	0.957	0.955	0.999	0.022	231
Non-*Anopheles*	0.590	0.833	0.837	0.999	0.087	630
*Drosophila*	0.590	0.874	0.869	0.999	0.072	435
Non-*Drosophila*	0.677	0.938	0.882	0.999	0.104	378
*Glossina*	0.965	0.994	0.986	0.999	0.014	15
Non-*Glossina*	0.441	0.739	0.772	0.999	0.144	1326
*Anopheles* vs. *Drosophila*	0.441	0.648	0.644	0.770	0.059	660
*Anopheles* vs. *Glossina*	0.677	0.740	0.744	0.787	0.027	132
*Drosophila* vs. *Glossina*	0.642	0.749	0.745	0.812	0.033	180
*C. briggsae* vs. *Anopheles*	0.528	0.559	0.562	0.643	0.030	22
*C. briggsae* vs. *Drosophila*	0.266	0.620	0.573	0.667	0.102	30
*C. briggsae* vs. *Glossina*	0.485	0.492	0.499	0.534	0.018	6
*A. mellifera* vs. *Anopheles*	0.568	0.617	0.629	0.702	0.043	22
*A. mellifera* vs. *Drosophila*	0.242	0.484	0.474	0.567	0.065	30
*A. mellifera* vs. *Glossina*	0.570	0.590	0.589	0.617	0.017	6

Octamers						
*Anopheles*	0.904	0.950	0.948	0.999	0.023	231
Non-*Anopheles*	0.588	0.824	0.822	0.998	0.089	630
*Drosophila*	0.588	0.858	0.857	0.997	0.069	435
Non-*Drosophila*	0.655	0.93	0.869	0.999	0.113	378
*Glossina*	0.948	0.988	0.978	0.998	0.020	15
Non-*Glossina*	0.443	0.723	0.761	0.999	0.143	1326
*Anopheles* vs. *Drosophila*	0.443	0.637	0.633	0.760	0.055	660
*Anopheles* vs. *Glossina*	0.655	0.716	0.719	0.755	0.026	132
*Drosophila* vs. *Glossina*	0.621	0.728	0.723	0.791	0.034	180
*C. briggsae* vs. *Anopheles*	0.521	0.554	0.556	0.634	0.029	22
*C. briggsae* vs. *Drosophila*	0.279	0.610	0.567	0.652	0.094	30
*C. briggsae* vs. *Glossina*	0.477	0.484	0.490	0.522	0.017	6
*A. mellifera* vs. *Anopheles*	0.564	0.611	0.624	0.696	0.042	22
*A. mellifera* vs. *Drosophila*	0.259	0.481	0.477	0.565	0.061	30
*A. mellifera* vs. *Glossina*	0.564	0.585	0.583	0.608	0.016	6

Nonamers						
*Anopheles*	0.886	0.939	0.938	0.996	0.025	231
Non-*Anopheles*	0.577	0.805	0.801	0.993	0.092	630
*Drosophila*	0.577	0.838	0.839	0.992	0.069	435
Non-*Drosophila*	0.629	0.919	0.852	0.996	0.121	378
*Glossina*	0.919	0.975	0.961	0.993	0.028	15
Non-*Glossina*	0.439	0.705	0.747	0.996	0.143	1326
*Anopheles* vs. *Drosophila*	0.439	0.624	0.619	0.746	0.053	660
*Anopheles* vs. *Glossina*	0.629	0.689	0.691	0.724	0.024	132
*Drosophila* vs. *Glossina*	0.589	0.697	0.694	0.766	0.034	180
*C. briggsae* vs. *Anopheles*	0.510	0.544	0.545	0.619	0.027	22
*C. briggsae* vs. *Drosophila*	0.285	0.594	0.553	0.636	0.086	30
*C. briggsae* vs. *Glossina*	0.464	0.470	0.475	0.503	0.014	6
*A. mellifera* vs. *Anopheles*	0.555	0.602	0.615	0.685	0.041	22
*A. mellifera* vs. *Drosophila*	0.270	0.475	0.474	0.558	0.058	30
*A. mellifera* vs. *Glossina*	0.551	0.572	0.570	0.592	0.014	6

CC values were calculated for the genera *Anopheles*, *Drosophila*, and *Glossina* as well as between these three genera and between two outliers, *Apis mellifera* and *Caenorhabditis elegans*, and these two genera. For each combination, the minimum, mean, median, maximum CC values were calculated as well as the standard deviation and the number of species comparisons.

**Table 3:** CC statistics for 5′, 3′ UTRs and introns for k-mer lengths *k* = 7–9 bp between *A. gambiae* and *Drosophila*.

Comparison	Region	k	Min	Median	Mean	Max	St. dev.	*n*	*p* value
Within *Drosophila* family^†^	5′ UTR	7	0.692	0.862	0.841	0.975	0.100	21	NA
*A. gambiae* vs. *Drosophila* family	5′ UTR	7	0.623	0.734	0.722	0.774	0.050	7	5.1*e*−4
Within *Drosophila* family	3′ UTR	7	0.651	0.828	0.809	0.963	0.101	21	NA
*A. gambiae* vs. *Drosophila* family	3′ UTR	7	0.524	0.620	0.599	0.644	0.043	7	6.2*e*−8
Within *Drosophila* family^‡^	Introns	7	0.759	0.894	0.895	0.996	0.058	66	NA
Within *Drosophila* family	5′ UTR	8	0.503	0.786	0.737	0.940	0.153	21	NA
*A. gambiae* vs. *Drosophila* family	5′ UTR	8	0.422	0.643	0.620	0.694	0.090	7	0.024
Within *Drosophila* family	3′ UTR	8	0.487	0.705	0.688	0.908	0.125	21	NA
*A. gambiae* vs. *Drosophila* family	3′ UTR	8	0.392	0.513	0.498	0.562	0.055	7	1.2*e*−5
Within *Drosophila* family	Introns	8	0.392	0.690	0.676	0.981	0.135	66	NA
Within *Drosophila* family	5′ UTR	9	0.280	0.626	0.569	0.854	0.183	21	NA
*A. gambiae* vs. *Drosophila* family	5′ UTR	9	0.201	0.453	0.431	0.512	0.104	7	0.023
Within *Drosophila* family	3′ UTR	9	0.334	0.526	0.524	0.795	0.122	21	NA
*A. gambiae* vs. *Drosophila* family	3′ UTR	9	0.242	0.360	0.356	0.422	0.056	7	5.9*e*−5
Within *Drosophila* family	Introns	9	0.721	0.855	0.854	0.978	0.062	66	NA

Minimum, mean, median, and maximum CC values were calculated for the 5′, 3′ UTR and intron regions of different *Drosophila* species compared to *A. gambiae*. The num[[parms resize(1),pos(50,50),size(200,200),bgcol(156)]] comparisons and the *p* value are also included. ^†^For 5′ and 3′ UTRs, the following *Drosophila* species were examined: *D. ananassae*, *erecta*, *grimshawi*, *melanogaster*, *mojavensis*, *pseudoobscura*, and *simulans*. ^‡^For introns, the following *Drosophila* species were examined: *D. ananassae*, *erecta*, *grimshawi*, *melanogaster*, *mojavensis*, *persimilis*, *pseudoobscura*, *sechelia*, *simulans*, *virilis*, *willistoni*, and *yakuba*.

## Data Availability

Processed data and results are available in the supplementary files. Raw genomic data will be made available upon request.

## Supplementary Materials

Supplementary Materials Supplemental Figure 1: genome size for all 58 studied species. The size of the genome of each species is given in Mbp. *Anopheles* species colored in blue, *Drosophila* species in red, and *Glossina* species in green. Supplemental Figure 2: ACGT% content for all 58 studied species. The ACGT% for all 58 species is given for all species, adding up to one in a stacked barplot. Supplemental Figure 3(a): heatmap depicting species relationships between the 63 species included in the analysis based on the whole-genome k-mer signature for heptamers. Supplemental Figure 3(b): heatmap depicting species relationships between the 63 species included in the analysis based on the whole-genome k-mer signature for nonamers. Supplemental Figure 4(a): Pearson correlation coefficient between species of *Anopheles*, *Drosophila*, and *Glossina* as well as the two control species, *A. mellifera* and *C. briggsae* for heptamers. Supplemental Figure 4(b): Pearson correlation coefficient between species of *Anopheles*, *Drosophila*, and *Glossina* as well as the two control species, *A. mellifera* and *C. briggsae* for nonamers. Supplemental Figure 5(a): common nonrepetitive (nondimer and nontrimer) heptamer content between 11 *Anopheles*, 15 *Drosophila*, and 5 *Glossina* species. Each included heptamer had a minimum score of 0.5. Supplemental Figure 5(b): common nonrepetitive (nondimer and nontrimer) nonamer content between 11 *Anopheles*, 15 *Drosophila* and 5 *Glossina* species. Each included nonamer had a minimum score of 0.5. Supplemental File 1: statistics of whole genome, 5′ and 3′ UTR, and intron sequences for the studied species. The species, file name, number of contigs, genome/subgenomic region size, and ACGT% are provided for each species. The pairwise sequence identity for all species pairs is included for the mitochondrial genome comparisons. Supplemental File 2: Pearson correlation matrix for whole-genome k-mer signatures. The Pearson correlation matrix between all pairs of the studied species is provided for k-mers of lengths 7–9 bp. Supplemental File 3: predicted biologically relevant whole-genome k-mers (octamers). Biologically relevant octamers were predicted by the k-mer prediction algorithm for the 22 *Anopheles*, 30 *Drosophila*, and six *Glossina* species. For the *Drosophila* species, all predicted octamers were matched against 140 *Drosophila* PWMs from the JASPAR database with a cutoff of 0.8. Supplemental File 4: high-scoring repetitive motif content of three *Drosophila* species. High-scoring and high-occurring octamer palindrome k-mers are listed for *Drosophila ananassae*, *mojavensis*, and *willistoni*. These k-mers were matched with the k-mers from other *Drosophila* species for comparison. Nonamers from *D. mojavensis* were also matched with motifs from the TRDB. Supplemental File 5: nonrepetitive frequent k-mers from the three genera. Nonrepetitive (nondimer/trimer repeats) were found in a majority of the species from the three genera for k-mers of lengths 7–9 bp. K-mers common to all three genera were also noted. Supplemental File 6: Pearson correlation matrix for 5′ UTR k-mer signatures. The Pearson correlation matrix between all pairs of species involving *A. gambiae* and seven *Drosophila* species is provided for k-mers of lengths 7–9 bp in the 5′ UTR regions. Common heptamers, octamers, and nonamers are also provided. Supplemental File 7: Pearson correlation matrix for 3′ UTR k-mer signatures. The Pearson correlation matrix between all pairs of species involving *A. gambiae* and seven *Drosophila* species is provided for k-mers of lengths 7–9 bp in the 3′ UTR regions. Common heptamers, octamers, and nonamers are also provided. Supplemental File 8: Pearson correlation matrix for intron k-mer signatures. The Pearson correlation matrix between all pairs of species involving 12 *Drosophila* species is provided for k-mers of lengths 7–9 bp in the intron regions. Common heptamers, octamers, and nonamers are also provided.
