# Supplementary figures and images for: K-mer-Based Motif Analysis in Insect Species across Anopheles, Drosophila, and Glossina Genera and Its Application to Species Classification
Source: Comput Math Methods Med. 2019 Nov 15;2019:4259479. doi: 10.1155/2019/4259479 (PMC6881769; doi:10.1155/2019/4259479)

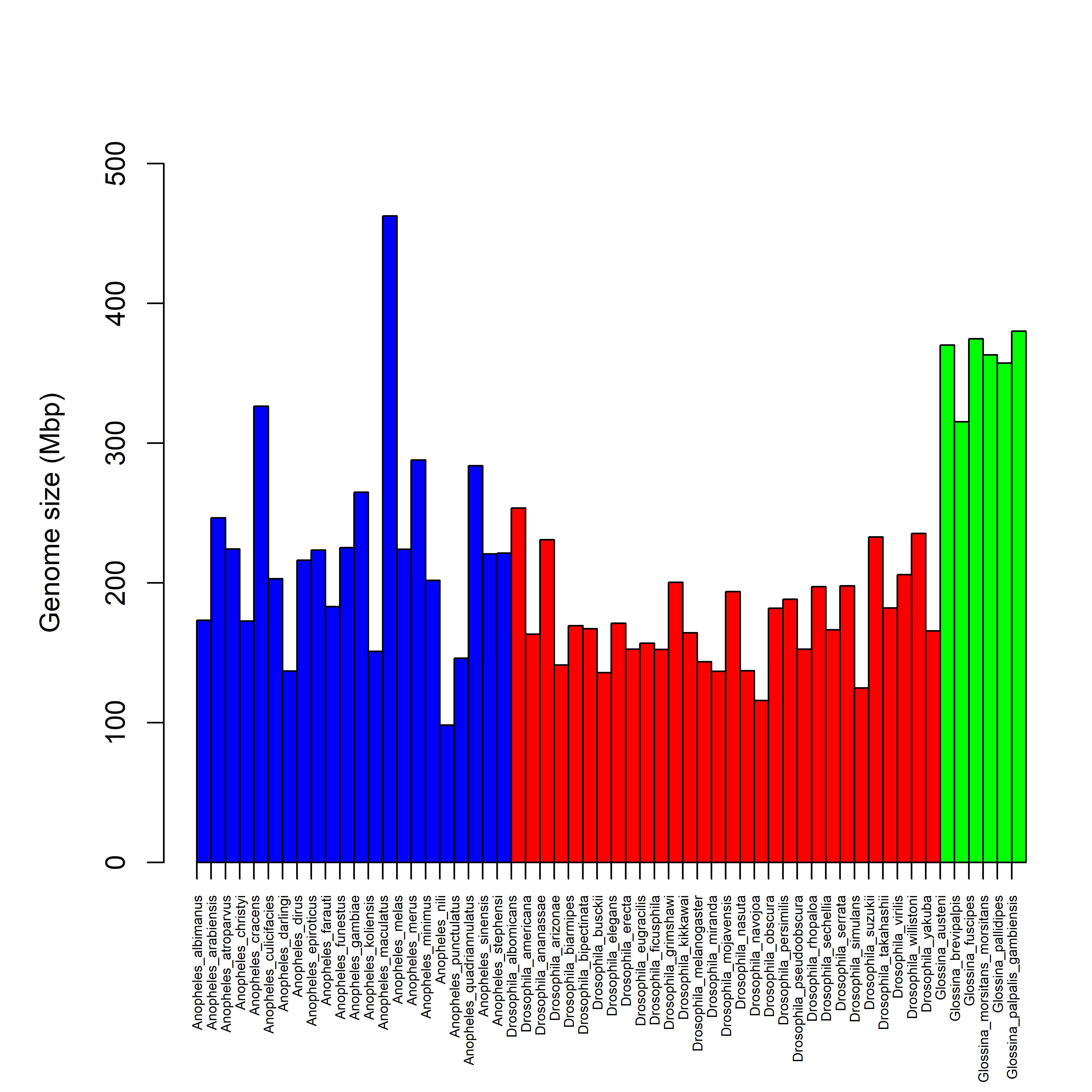

Supplement: Supplementary Materials — Supplemental Figure 1: genome size for all 58 studied species. The size of the genome of each species is given in Mbp. Anopheles species colored in blue, Drosophila species in red, and Glossina species in green. Supplemental Figure 2: ACGT% content for all 58 studied species. The ACGT% for all 58 species is given for all species, adding up to one in a stacked barplot. Supplemental Figure 3(a): heatmap depicting species relationships between the 63 species included in the analysis based on the whole-genome k-mer signature for heptamers. Supplemental Figure 3(b): heatmap depicting species relationships between the 63 species included in the analysis based on the whole-genome k-mer signature for nonamers. Supplemental Figure 4(a): Pearson correlation coefficient between species of Anopheles, Drosophila, and Glossina as well as the two control species, A. mellifera and C. briggsae for heptamers. Supplemental Figure 4(b): Pearson correlation coefficient between species of Anopheles, Drosophila, and Glossina as well as the two control species, A. mellifera and C. briggsae for nonamers. Supplemental Figure 5(a): common nonrepetitive (nondimer and nontrimer) heptamer content between 11 Anopheles, 15 Drosophila, and 5 Glossina species. Each included heptamer had a minimum score of 0.5. Supplemental Figure 5(b): common nonrepetitive (nondimer and nontrimer) nonamer content between 11 Anopheles, 15 Drosophila and 5 Glossina species. Each included nonamer had a minimum score of 0.5. Supplemental File 1: statistics of whole genome, 5′ and 3′ UTR, and intron sequences for the studied species. The species, file name, number of contigs, genome/subgenomic region size, and ACGT% are provided for each species. The pairwise sequence identity for all species pairs is included for the mitochondrial genome comparisons. Supplemental File 2: Pearson correlation matrix for whole-genome k-mer signatures. The Pearson correlation matrix between all pairs of the studied species is provided for k [file 4259479.f1.zip › 4259479.f1/S1_Fig.tiff]

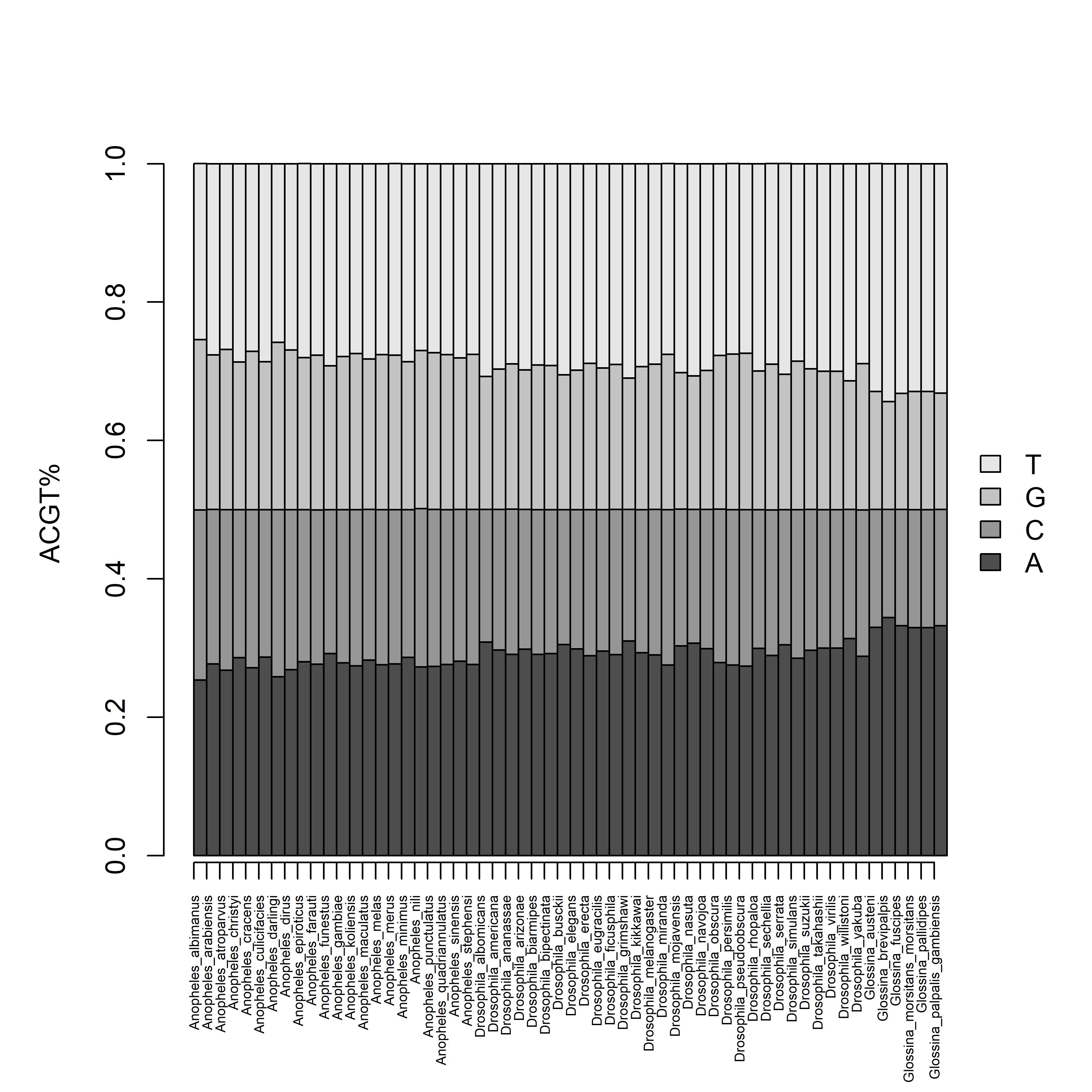

Supplement: Supplementary Materials — Supplemental Figure 1: genome size for all 58 studied species. The size of the genome of each species is given in Mbp. Anopheles species colored in blue, Drosophila species in red, and Glossina species in green. Supplemental Figure 2: ACGT% content for all 58 studied species. The ACGT% for all 58 species is given for all species, adding up to one in a stacked barplot. Supplemental Figure 3(a): heatmap depicting species relationships between the 63 species included in the analysis based on the whole-genome k-mer signature for heptamers. Supplemental Figure 3(b): heatmap depicting species relationships between the 63 species included in the analysis based on the whole-genome k-mer signature for nonamers. Supplemental Figure 4(a): Pearson correlation coefficient between species of Anopheles, Drosophila, and Glossina as well as the two control species, A. mellifera and C. briggsae for heptamers. Supplemental Figure 4(b): Pearson correlation coefficient between species of Anopheles, Drosophila, and Glossina as well as the two control species, A. mellifera and C. briggsae for nonamers. Supplemental Figure 5(a): common nonrepetitive (nondimer and nontrimer) heptamer content between 11 Anopheles, 15 Drosophila, and 5 Glossina species. Each included heptamer had a minimum score of 0.5. Supplemental Figure 5(b): common nonrepetitive (nondimer and nontrimer) nonamer content between 11 Anopheles, 15 Drosophila and 5 Glossina species. Each included nonamer had a minimum score of 0.5. Supplemental File 1: statistics of whole genome, 5′ and 3′ UTR, and intron sequences for the studied species. The species, file name, number of contigs, genome/subgenomic region size, and ACGT% are provided for each species. The pairwise sequence identity for all species pairs is included for the mitochondrial genome comparisons. Supplemental File 2: Pearson correlation matrix for whole-genome k-mer signatures. The Pearson correlation matrix between all pairs of the studied species is provided for k [file 4259479.f1.zip › 4259479.f1/S2_Fig.tiff]

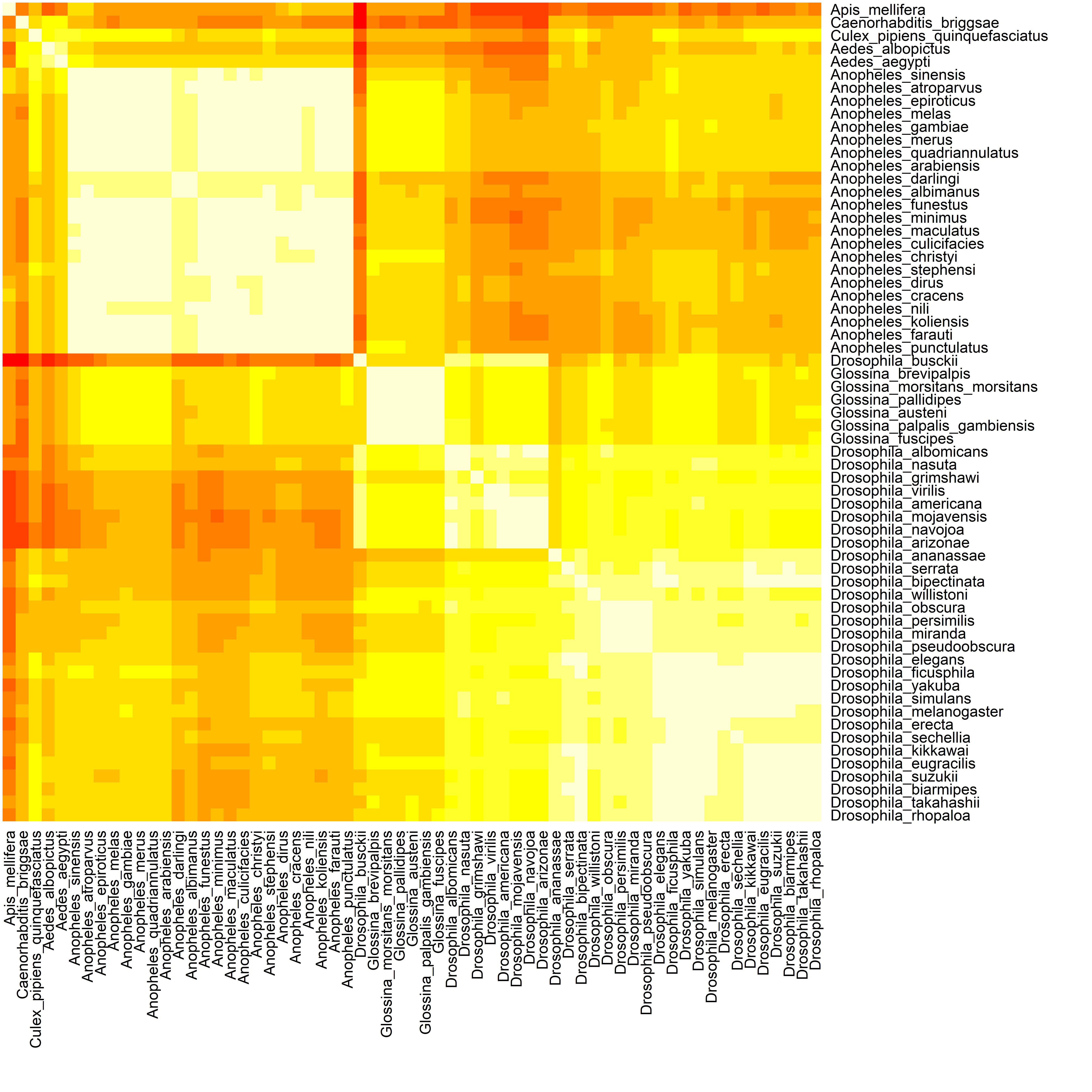

Supplement: Supplementary Materials — Supplemental Figure 1: genome size for all 58 studied species. The size of the genome of each species is given in Mbp. Anopheles species colored in blue, Drosophila species in red, and Glossina species in green. Supplemental Figure 2: ACGT% content for all 58 studied species. The ACGT% for all 58 species is given for all species, adding up to one in a stacked barplot. Supplemental Figure 3(a): heatmap depicting species relationships between the 63 species included in the analysis based on the whole-genome k-mer signature for heptamers. Supplemental Figure 3(b): heatmap depicting species relationships between the 63 species included in the analysis based on the whole-genome k-mer signature for nonamers. Supplemental Figure 4(a): Pearson correlation coefficient between species of Anopheles, Drosophila, and Glossina as well as the two control species, A. mellifera and C. briggsae for heptamers. Supplemental Figure 4(b): Pearson correlation coefficient between species of Anopheles, Drosophila, and Glossina as well as the two control species, A. mellifera and C. briggsae for nonamers. Supplemental Figure 5(a): common nonrepetitive (nondimer and nontrimer) heptamer content between 11 Anopheles, 15 Drosophila, and 5 Glossina species. Each included heptamer had a minimum score of 0.5. Supplemental Figure 5(b): common nonrepetitive (nondimer and nontrimer) nonamer content between 11 Anopheles, 15 Drosophila and 5 Glossina species. Each included nonamer had a minimum score of 0.5. Supplemental File 1: statistics of whole genome, 5′ and 3′ UTR, and intron sequences for the studied species. The species, file name, number of contigs, genome/subgenomic region size, and ACGT% are provided for each species. The pairwise sequence identity for all species pairs is included for the mitochondrial genome comparisons. Supplemental File 2: Pearson correlation matrix for whole-genome k-mer signatures. The Pearson correlation matrix between all pairs of the studied species is provided for k [file 4259479.f1.zip › 4259479.f1/S3a_Fig.tiff]

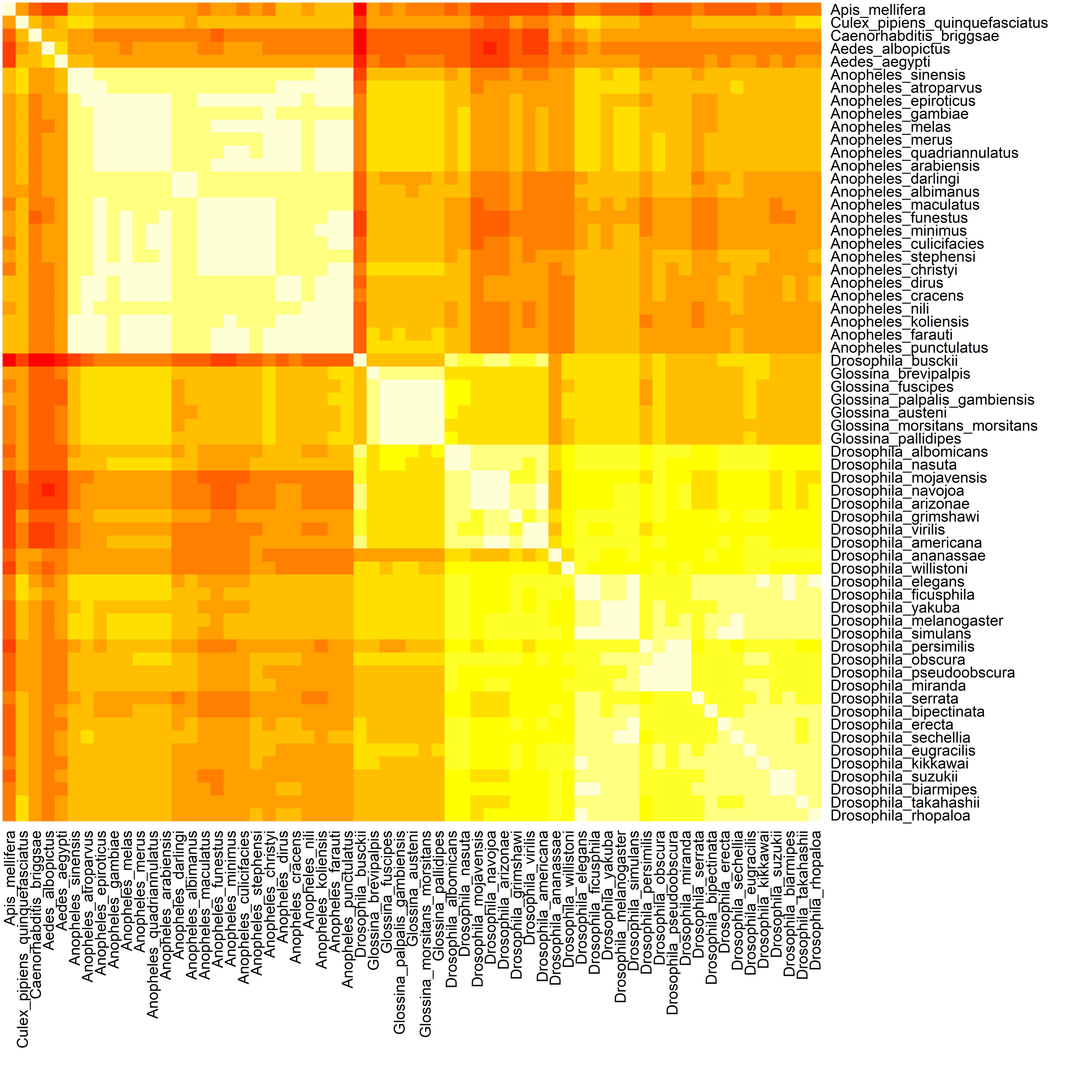

Supplement: Supplementary Materials — Supplemental Figure 1: genome size for all 58 studied species. The size of the genome of each species is given in Mbp. Anopheles species colored in blue, Drosophila species in red, and Glossina species in green. Supplemental Figure 2: ACGT% content for all 58 studied species. The ACGT% for all 58 species is given for all species, adding up to one in a stacked barplot. Supplemental Figure 3(a): heatmap depicting species relationships between the 63 species included in the analysis based on the whole-genome k-mer signature for heptamers. Supplemental Figure 3(b): heatmap depicting species relationships between the 63 species included in the analysis based on the whole-genome k-mer signature for nonamers. Supplemental Figure 4(a): Pearson correlation coefficient between species of Anopheles, Drosophila, and Glossina as well as the two control species, A. mellifera and C. briggsae for heptamers. Supplemental Figure 4(b): Pearson correlation coefficient between species of Anopheles, Drosophila, and Glossina as well as the two control species, A. mellifera and C. briggsae for nonamers. Supplemental Figure 5(a): common nonrepetitive (nondimer and nontrimer) heptamer content between 11 Anopheles, 15 Drosophila, and 5 Glossina species. Each included heptamer had a minimum score of 0.5. Supplemental Figure 5(b): common nonrepetitive (nondimer and nontrimer) nonamer content between 11 Anopheles, 15 Drosophila and 5 Glossina species. Each included nonamer had a minimum score of 0.5. Supplemental File 1: statistics of whole genome, 5′ and 3′ UTR, and intron sequences for the studied species. The species, file name, number of contigs, genome/subgenomic region size, and ACGT% are provided for each species. The pairwise sequence identity for all species pairs is included for the mitochondrial genome comparisons. Supplemental File 2: Pearson correlation matrix for whole-genome k-mer signatures. The Pearson correlation matrix between all pairs of the studied species is provided for k [file 4259479.f1.zip › 4259479.f1/S3b_Fig.tiff]

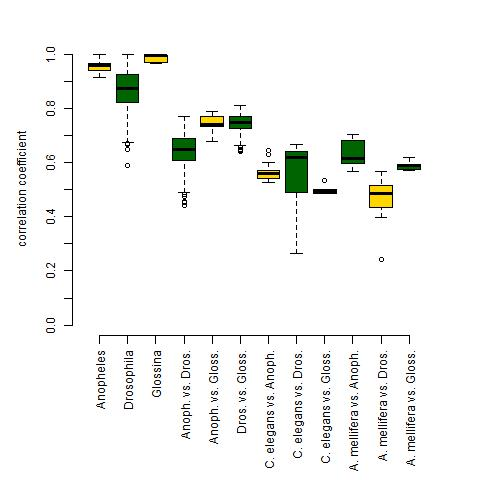

Supplement: Supplementary Materials — Supplemental Figure 1: genome size for all 58 studied species. The size of the genome of each species is given in Mbp. Anopheles species colored in blue, Drosophila species in red, and Glossina species in green. Supplemental Figure 2: ACGT% content for all 58 studied species. The ACGT% for all 58 species is given for all species, adding up to one in a stacked barplot. Supplemental Figure 3(a): heatmap depicting species relationships between the 63 species included in the analysis based on the whole-genome k-mer signature for heptamers. Supplemental Figure 3(b): heatmap depicting species relationships between the 63 species included in the analysis based on the whole-genome k-mer signature for nonamers. Supplemental Figure 4(a): Pearson correlation coefficient between species of Anopheles, Drosophila, and Glossina as well as the two control species, A. mellifera and C. briggsae for heptamers. Supplemental Figure 4(b): Pearson correlation coefficient between species of Anopheles, Drosophila, and Glossina as well as the two control species, A. mellifera and C. briggsae for nonamers. Supplemental Figure 5(a): common nonrepetitive (nondimer and nontrimer) heptamer content between 11 Anopheles, 15 Drosophila, and 5 Glossina species. Each included heptamer had a minimum score of 0.5. Supplemental Figure 5(b): common nonrepetitive (nondimer and nontrimer) nonamer content between 11 Anopheles, 15 Drosophila and 5 Glossina species. Each included nonamer had a minimum score of 0.5. Supplemental File 1: statistics of whole genome, 5′ and 3′ UTR, and intron sequences for the studied species. The species, file name, number of contigs, genome/subgenomic region size, and ACGT% are provided for each species. The pairwise sequence identity for all species pairs is included for the mitochondrial genome comparisons. Supplemental File 2: Pearson correlation matrix for whole-genome k-mer signatures. The Pearson correlation matrix between all pairs of the studied species is provided for k [file 4259479.f1.zip › 4259479.f1/S4a_Fig.tiff]

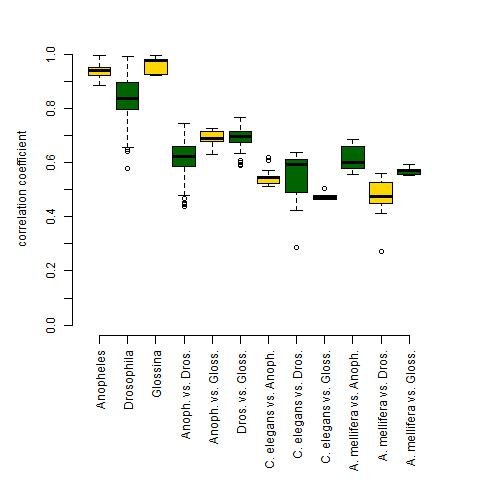

Supplement: Supplementary Materials — Supplemental Figure 1: genome size for all 58 studied species. The size of the genome of each species is given in Mbp. Anopheles species colored in blue, Drosophila species in red, and Glossina species in green. Supplemental Figure 2: ACGT% content for all 58 studied species. The ACGT% for all 58 species is given for all species, adding up to one in a stacked barplot. Supplemental Figure 3(a): heatmap depicting species relationships between the 63 species included in the analysis based on the whole-genome k-mer signature for heptamers. Supplemental Figure 3(b): heatmap depicting species relationships between the 63 species included in the analysis based on the whole-genome k-mer signature for nonamers. Supplemental Figure 4(a): Pearson correlation coefficient between species of Anopheles, Drosophila, and Glossina as well as the two control species, A. mellifera and C. briggsae for heptamers. Supplemental Figure 4(b): Pearson correlation coefficient between species of Anopheles, Drosophila, and Glossina as well as the two control species, A. mellifera and C. briggsae for nonamers. Supplemental Figure 5(a): common nonrepetitive (nondimer and nontrimer) heptamer content between 11 Anopheles, 15 Drosophila, and 5 Glossina species. Each included heptamer had a minimum score of 0.5. Supplemental Figure 5(b): common nonrepetitive (nondimer and nontrimer) nonamer content between 11 Anopheles, 15 Drosophila and 5 Glossina species. Each included nonamer had a minimum score of 0.5. Supplemental File 1: statistics of whole genome, 5′ and 3′ UTR, and intron sequences for the studied species. The species, file name, number of contigs, genome/subgenomic region size, and ACGT% are provided for each species. The pairwise sequence identity for all species pairs is included for the mitochondrial genome comparisons. Supplemental File 2: Pearson correlation matrix for whole-genome k-mer signatures. The Pearson correlation matrix between all pairs of the studied species is provided for k [file 4259479.f1.zip › 4259479.f1/S4b_Fig.tiff]

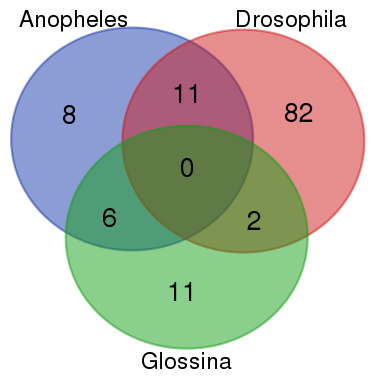

Supplement: Supplementary Materials — Supplemental Figure 1: genome size for all 58 studied species. The size of the genome of each species is given in Mbp. Anopheles species colored in blue, Drosophila species in red, and Glossina species in green. Supplemental Figure 2: ACGT% content for all 58 studied species. The ACGT% for all 58 species is given for all species, adding up to one in a stacked barplot. Supplemental Figure 3(a): heatmap depicting species relationships between the 63 species included in the analysis based on the whole-genome k-mer signature for heptamers. Supplemental Figure 3(b): heatmap depicting species relationships between the 63 species included in the analysis based on the whole-genome k-mer signature for nonamers. Supplemental Figure 4(a): Pearson correlation coefficient between species of Anopheles, Drosophila, and Glossina as well as the two control species, A. mellifera and C. briggsae for heptamers. Supplemental Figure 4(b): Pearson correlation coefficient between species of Anopheles, Drosophila, and Glossina as well as the two control species, A. mellifera and C. briggsae for nonamers. Supplemental Figure 5(a): common nonrepetitive (nondimer and nontrimer) heptamer content between 11 Anopheles, 15 Drosophila, and 5 Glossina species. Each included heptamer had a minimum score of 0.5. Supplemental Figure 5(b): common nonrepetitive (nondimer and nontrimer) nonamer content between 11 Anopheles, 15 Drosophila and 5 Glossina species. Each included nonamer had a minimum score of 0.5. Supplemental File 1: statistics of whole genome, 5′ and 3′ UTR, and intron sequences for the studied species. The species, file name, number of contigs, genome/subgenomic region size, and ACGT% are provided for each species. The pairwise sequence identity for all species pairs is included for the mitochondrial genome comparisons. Supplemental File 2: Pearson correlation matrix for whole-genome k-mer signatures. The Pearson correlation matrix between all pairs of the studied species is provided for k [file 4259479.f1.zip › 4259479.f1/S5a_Fig.tiff]

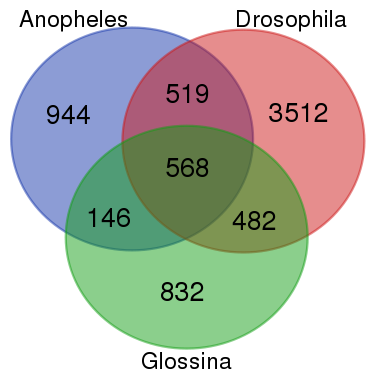

Supplement: Supplementary Materials — Supplemental Figure 1: genome size for all 58 studied species. The size of the genome of each species is given in Mbp. Anopheles species colored in blue, Drosophila species in red, and Glossina species in green. Supplemental Figure 2: ACGT% content for all 58 studied species. The ACGT% for all 58 species is given for all species, adding up to one in a stacked barplot. Supplemental Figure 3(a): heatmap depicting species relationships between the 63 species included in the analysis based on the whole-genome k-mer signature for heptamers. Supplemental Figure 3(b): heatmap depicting species relationships between the 63 species included in the analysis based on the whole-genome k-mer signature for nonamers. Supplemental Figure 4(a): Pearson correlation coefficient between species of Anopheles, Drosophila, and Glossina as well as the two control species, A. mellifera and C. briggsae for heptamers. Supplemental Figure 4(b): Pearson correlation coefficient between species of Anopheles, Drosophila, and Glossina as well as the two control species, A. mellifera and C. briggsae for nonamers. Supplemental Figure 5(a): common nonrepetitive (nondimer and nontrimer) heptamer content between 11 Anopheles, 15 Drosophila, and 5 Glossina species. Each included heptamer had a minimum score of 0.5. Supplemental Figure 5(b): common nonrepetitive (nondimer and nontrimer) nonamer content between 11 Anopheles, 15 Drosophila and 5 Glossina species. Each included nonamer had a minimum score of 0.5. Supplemental File 1: statistics of whole genome, 5′ and 3′ UTR, and intron sequences for the studied species. The species, file name, number of contigs, genome/subgenomic region size, and ACGT% are provided for each species. The pairwise sequence identity for all species pairs is included for the mitochondrial genome comparisons. Supplemental File 2: Pearson correlation matrix for whole-genome k-mer signatures. The Pearson correlation matrix between all pairs of the studied species is provided for k [file 4259479.f1.zip › 4259479.f1/S5b_Fig.tiff]
